# Night eating syndrome in women with gestational diabetes mellitus: a comparative cross-sectional study

**DOI:** 10.1590/1806-9282.20260783

**Published:** 2026-10-05

**Authors:** Esra Keles, Leyla Kaya, Cansu Ergenç Özdaş, Zahide Kaya

**Affiliations:** 1 University of Health Sciences, Kartal Dr. Lütfi Kırdar City Hospital, Department of Gynecologic Oncology – Istanbul, Turkey.; 2 University of Health Sciences, Faculty of Health Sciences, Department of Midwifery – Istanbul, Turkey.; 3 Ankara Yildirim Beyazit University, Business School – Ankara, Turkey.; 4 Uskudar State Hospital, Internal Medicine Clinic – Istanbul, Turkey.

**Keywords:** Diabetes, Gestational, Feeding and eating disorders, Sleep quality, Fatigue, Night eating syndrome

## Abstract

**OBJECTIVE:**

The aim of this study was to determine the prevalence of night eating syndrome among women with gestational diabetes mellitus compared with healthy pregnant controls, and to evaluate its associations with sleep quality, healthy lifestyle behaviors, fatigue severity, physical activity, perceived social support, and neonatal outcomes.

**METHODS:**

This single-center, comparative cross-sectional study enrolled 615 pregnant women at a tertiary hospital. Night eating syndrome was identified using the Night Eating Questionnaire ≥25. Sleep quality, lifestyle behaviors, fatigue, physical activity, and perceived social support were assessed with the Pittsburgh Sleep Quality Index, Healthy Lifestyle Behaviors Scale, Fatigue Severity Scale, International Physical Activity Questionnaire, and Multidimensional Scale of Perceived Social Support. Group comparisons, and multivariable logistic regression with Benjamini-Hochberg correction were performed.

**RESULTS:**

Night eating syndrome prevalence was higher in women with gestational diabetes mellitus than in controls (p<0.001). The gestational diabetes mellitus group exhibited worse sleep (Pittsburgh Sleep Quality Index 17.2±4.0 vs. 8.3±5.7), lower Healthy Lifestyle Behaviors Scale scores, greater fatigue, and reduced social support (all p<0.001). Higher Healthy Lifestyle Behaviors Scale and physical activity were protective against night eating syndrome. Night eating syndrome was independently associated with a nearly fourfold increase in neonatal intensive care unit admission (adjusted odds ratio 3.82; 95%CI 1.68–8.71; p=0.001) after adjustment for gestational diabetes mellitus, body mass index, sleep, fatigue, lifestyle, gestational age at birth, and maternal age.

**CONCLUSION:**

Night eating syndrome is highly prevalent among women with gestational diabetes mellitus, clustering with poor sleep, severe fatigue, sedentariness, and reduced social support, and independently associated with adverse neonatal outcomes.

## INTRODUCTION

Gestational diabetes mellitus (GDM) is among the most common metabolic complications of pregnancy, with a global prevalence ranging from 5 to 20% depending on diagnostic thresholds and population characteristics^1^. Its clinical burden extends beyond maternal hyperglycemia and encompasses preeclampsia, macrosomia, neonatal hypoglycemia, birth trauma, and increased neonatal intensive care unit (NICU) admission^2,3^. Although current guidelines emphasize medical nutrition therapy, structured physical activity, glucose self-monitoring, and pharmacotherapy when indicated, the behavioral and psychosocial dimensions of GDM remain comparatively underexplored^4^.

Night eating syndrome (NES) is a circadian-related eating disorder characterized by evening hyperphagia, nocturnal ingestion, morning anorexia, and concurrent sleep disturbance^5^. NES is classified within the Diagnostic and Statistical Manual of Mental Disorders, Fifth Edition (DSM-5) category of “Other Specified Feeding or Eating Disorders,” and the Night Eating Questionnaire (NEQ) is the most widely validated screening instrument^6^. While general-population prevalence is 1.5–5%, rates rise to 6–16% in obesity and exceed 25% among bariatric surgery candidates^7,8^. Proposed mechanisms include circadian misalignment, hypothalamic–pituitary–adrenal axis dysregulation, and altered melatonin–leptin–ghrelin signaling—pathways that plausibly intersect with the metabolic disturbances of GDM^9,10^.

Despite this biological plausibility, NES in GDM has received limited systematic attention. Existing research on disordered eating in pregnancy has focused predominantly on binge eating and bulimia nervosa^11,12^, leaving the prevalence, behavioral clustering, and neonatal implications of NES in GDM largely undefined. Furthermore, whether NES contributes independently to neonatal morbidity—beyond conventional GDM-related risk pathways has not been adequately examined^13,14^.

Accordingly, this study aimed to (i) compare NES prevalence between women with GDM and healthy pregnant controls; (ii) characterize the psychobehavioral and clinical profile associated with NES; and (iii) identify independent predictors of NES.

## METHODS

### Study design

A single-center, comparative cross-sectional study was conducted at a tertiary obstetric referral center. The investigation conformed to the Declaration of Helsinki and was reported in accordance with the Strengthening the Reporting of Observational Studies in Epidemiology (STROBE) statement.

### Participants

Women diagnosed with GDM by the International Association of Diabetes and Pregnancy Study Groups criteria and healthy pregnant controls were consecutively enrolled during routine antenatal visits.

### Inclusion and exclusion criteria

Inclusion criteria were singleton pregnancy, maternal age ≥18 years, gestational age ≥24 weeks, and ability to complete self-report instruments in the study language. Exclusion criteria included pre-existing type 1 or type 2 diabetes mellitus, multiple gestations, psychiatric disorders requiring pharmacotherapy, and major fetal anomalies. The final sample comprised 615 women (271 GDM; 344 healthy controls).

### Measurements and scales

Sociodemographic, obstetric, and anthropometric data (maternal age, gravidity, parity, planning status, gestational age at assessment, pre-pregnancy and current weight, body mass index [BMI], gestational weight gain) were retrieved from medical records and verified by structured interview.

NES was assessed using the 14-item NEQ (range 0–52); a score ≥25 defined clinically significant NES^15^. Sleep quality was measured using the Pittsburgh Sleep Quality Index (PSQI; range 0–21), with scores>5 indicating poor sleep^16^. Fatigue was evaluated by the Fatigue Severity Scale (FSS; range 1–7), with a mean score ≥4 defining severe fatigue^17^. Health-promoting behaviors were measured by the Healthy Lifestyle Behaviors Scale (HLBS; range 29–145)^18^. Perceived social support was assessed by the Multidimensional Scale of Perceived Social Support (MSPSS; range 12–60)^19^, and habitual physical activity by the International Physical Activity Questionnaire (IPAQ)^20^. Gestational age at birth, birth weight, 1- and 5-min Apgar scores, NICU admission, and respiratory support were extracted from delivery and neonatal records.

### Statistical analysis

Distributions were tested by the Shapiro-Wilk test. Continuous variables were expressed as mean±standard deviation and compared using the Mann-Whitney U test owing to non-normal distributions; categorical variables were compared using the chi-square test. Effect sizes were reported as Cohen’s d for continuous variables and Cramér’s V (φ) for categorical variables. Two multivariable binary logistic regression models were constructed: Model A predicting NES (NEQ≥25) and Model B predicting NICU admission. Covariates were selected a priori based on clinical relevance and bivariate associations. Model performance was evaluated by the Hosmer-Lemeshow goodness-of-fit test, Nagelkerke pseudo-R^2^, and classification accuracy. The Benjamini-Hochberg false discovery rate correction was applied across the behavioral cluster (q<0.05). Two-sided p<0.05 was considered statistically significant. Analyses were performed using Statistical Package for the Social Sciences v26.0 (IBM Corp., Armonk, NY, USA).

## RESULTS

### Baseline characteristics

Women with GDM were significantly older, of higher gravidity, and had greater pre-pregnancy and current pregnancy weight than controls (all p<0.001; Table 1). They were also assessed earlier in gestation (38.1±1.9 vs. 38.9±1.7 weeks; p<0.001). Gestational age at birth was lower in the GDM group (38.1±1.9 vs. 39.9±1.8 weeks; p<0.001; d=0.97), birth weight was higher (p=0.015), and 1- and 5-min Apgar scores were lower (p<0.001). NICU admission was more frequent in the GDM cohort (18.8 vs. 10.2%; p=0.003; φ=0.12).

**Table 1.** Baseline sociodemographic, obstetric, anthropometric, and neonatal characteristics of women with gestational diabetes mellitus versus healthy pregnant controls (n=615).

Characteristic	GDM (n=271)	Controls (n=344)	p-value†
Maternal age (years)	31.5±5.8	28.2±4.9	<0.001
Gravidity	2.8±1.6	2.1±1.3	<0.001
Gestational age at assessment (weeks)	38.1±1.9	38.9±1.7	<0.001
Pre-pregnancy weight (kg)	72.0±14.1	64.1±11.4	<0.001
Current pregnancy weight (kg)	83.8±13.6	78.8±11.4	<0.001
PSQI total score	17.2±4.0	8.3±5.7	<0.001
HLBS total score	105.7±7.2	128.1±3.7	<0.001
FSS mean score	4.26±1.32	2.28±1.11	<0.001
Gestational age at birth (weeks)	38.1±1.9	39.9±1.8	<0.001
Birth weight (g)	3325±538	3258±490	0.015
Apgar 1-min	7.28±1.03	7.60±0.74	<0.001
Apgar 5-min	8.68±0.62	8.83±0.49	<0.001
NICU admission, n (%)	51 (18.8)	35 (10.2)	0.003

†Mann-Whitney U for continuous; χ^2^ for categorical. Data are mean±SD unless otherwise indicated. FSS: Fatigue Severity Scale; GDM: gestational diabetes mellitus; HLBS: Healthy Lifestyle Behaviors Scale; NICU: neonatal intensive care unit; PSQI: Pittsburgh Sleep Quality Index; SD: standard deviation.

### Prevalence of night eating syndrome and group differences in psychobehavioral profile

NES (NEQ≥25) was identified in 83/615 (13.5%) of the overall cohort. Among women with GDM, 78/271 (28.8%) met NES criteria compared with 5/344 (1.5%) of controls (p<0.001; φ=0.49). Women with GDM demonstrated significantly poorer sleep quality (PSQI 17.2±4.0 vs. 8.3±5.7), lower HLBS scores (105.7±7.2 vs. 128.1±3.7), greater fatigue (FSS 4.26±1.32 vs. 2.28±1.11), and reduced social support (all p<0.001).

### Psychobehavioral and neonatal profiles by night eating syndrome status

NES-positive women did not differ from NES-negative women in maternal age but had higher pre-pregnancy weight (76.5±15.1 vs. 70.2±13.4 kg; p<0.001) and BMI (28.9±5.9 vs. 27.3±5.5 kg/m^2^; p=0.018; Table 2). PSQI scores were markedly higher among NES-positive women (18.5±4.1 vs. 11.8±6.3; d=1.16), and all NES-positive participants exceeded the poor-sleep threshold (PSQI>5: 100 vs. 75.8%; p<0.001). The largest behavioral effect size was observed for fatigue (FSS 4.92±1.18 vs. 2.86±1.36; d=1.55), with severe fatigue (FSS≥4) present in 81.9 vs. 20.9% (p<0.001). HLBS (d=0.97), physical activity (d=0.36), and MSPSS (d=0.67) scores were significantly lower in the NES-positive group.

**Table 2.** Behavioral, psychosocial, and neonatal profiles stratified by night eating syndrome status (n=615).

Variable	NES+ (NEQ≥25) (n=83)	NES- (NEQ<25) (n=532)	p-value†
GDM, n (%)	78 (94.0)	193 (36.3)	<0.001
Maternal age (years)	31.2±6.4	31.6±5.5	0.527
Pre-pregnancy weight (kg)	76.5±15.1	70.2±13.4	<0.001
Pre-pregnancy BMI (kg/m^2^)	28.9±5.9	27.3±5.5	0.018
Gestational weight gain (kg)	13.8±6.5	13.3±6.0	0.420
NEQ total (0–52)	29.1±4.2	12.4±6.9	<0.001
PSQI total (0–21)	18.5±4.1	11.8±6.3	<0.001
Poor sleep (PSQI>5), n (%)	83 (100.0)	403 (75.8)	<0.001
HLBS total (29–145)	104.1±6.5	116.8±14.7	<0.001
FSS mean (1–7)	4.92±1.18	2.86±1.36	<0.001
Severe fatigue (FSS≥4), n (%)	68 (81.9)	111 (20.9)	<0.001
IPAQ score	22.6±14.8	28.4±16.2	0.003
MSPSS (12–60)	46.7±12.0	54.9±12.3	<0.001
Gestational age at birth (weeks)	37.9±2.2	38.2±1.7	0.231
Birth weight (g)	3417±647	3288±484	0.081
Apgar 1-min	7.10±1.00	7.35±1.04	0.026
Apgar 5-min	8.64±0.60	8.70±0.62	0.276
NICU admission, n (%)	26 (31.3)	60 (11.3)	<0.001
Respiratory support, n (%)	26 (31.3)	60 (11.3)	<0.001

†Mann-Whitney U/χ^2^. Reported p-values are unadjusted; Benjamini-Hochberg FDR correction applied across the behavioral cluster (q<0.05 for all significant findings). BMI: body mass index; FSS: Fatigue Severity Scale; GDM: gestational diabetes mellitus; HLBS: Healthy Lifestyle Behaviors Scale; IPAQ: International Physical Activity Questionnaire; MSPSS: Multidimensional Scale of Perceived Social Support; NEQ: Night Eating Questionnaire; NES: night eating syndrome; NICU: neonatal intensive care unit; PSQI: Pittsburgh Sleep Quality Index.

Neonates of NES-positive mothers had lower 1-min Apgar scores (7.10±1.00 vs. 7.35±1.04; p=0.026), although gestational age at birth, birth weight, and 5-min Apgar scores did not differ. NICU admission was nearly threefold more common in the NES-positive group (31.3 vs. 11.3%; p<0.001; φ=0.21).

### Multivariable analyses

In Model A (Table 3), GDM was the strongest independent predictor of NES (adjusted odds ratio [AOR] 6.42; 95%CI 3.10–13.30; p<0.001). Fatigue (AOR 1.42), pre-pregnancy BMI (AOR 1.08), PSQI (AOR 1.07), and unplanned pregnancy (AOR 1.69) were also independently associated with NES, whereas higher HLBS (AOR 0.96) and physical activity (AOR 0.98) scores were protective. Model calibration (Hosmer-Lemeshow p=0.45) and discrimination (Nagelkerke R^2^=0.31; classification accuracy 78.4%) were adequate.

**Table 3.** Multivariable logistic regression: independent predictors of (A) night eating syndrome and (B) neonatal intensive care unit admission.

Predictor	AOR	95%CI	p-value
Model A—Outcome: NES (NEQ≥25)			
GDM status (vs. control)	6.42	3.10–13.30	<0.001
Pre-pregnancy BMI (per kg/m^2^)	1.08	1.02–1.14	0.008
PSQI score (per point)	1.07	1.01–1.14	0.028
FSS score (per point)	1.42	1.18–1.71	<0.001
HLBS score (per point)	0.96	0.93–0.99	0.009
IPAQ score (per point)	0.98	0.96–1.00	0.034
Unplanned pregnancy (vs. planned)	1.69	1.01–2.82	0.045
Maternal age (per year)	1.02	0.96–1.08	0.535
Model fit: Nagelkerke R^2^=0.31; Hosmer-Lemeshow p=0.45; accuracy=78.4%			
**Model B—Outcome: NICU admission**			
NES (NEQ≥25)	3.82	1.68–8.71	0.001
GDM status (vs. control)	1.94	1.18–3.18	0.009
Pre-pregnancy BMI (per kg/m^2^)	1.05	1.00–1.10	0.062
PSQI score (per point)	1.03	0.99–1.07	0.140
FSS score (per point)	1.12	0.94–1.34	0.205
HLBS score (per point)	1.01	0.98–1.04	0.512
Gestational age at birth (per week)	0.71	0.60–0.84	<0.001
Maternal age (per year)	0.99	0.94–1.04	0.621
Model fit: events=86; Nagelkerke R^2^=0.22; Hosmer-Lemeshow p=0.38; LR χ^2^=38.6, p<0.001			

AOR: adjusted odds ratio; BMI: body mass index; CI: confidence interval; FSS: Fatigue Severity Scale; GDM: gestational diabetes mellitus; HLBS: Healthy Lifestyle Behaviors Scale; IPAQ: International Physical Activity Questionnaire; LR: likelihood ratio; NEQ: Night Eating Questionnaire; NES: night eating syndrome; NICU: neonatal intensive care unit; PSQI: Pittsburgh Sleep Quality Index.

In Model B, NES was independently associated with NICU admission (AOR 3.82; 95%CI 1.68–8.71; p=0.001) after adjustment for GDM status, BMI, sleep quality, fatigue, lifestyle behaviors, gestational age at birth, and maternal age. GDM (AOR 1.94; p=0.009) and gestational age at birth (AOR 0.71 per week; p<0.001) were also significant predictors. Model fit was acceptable (Nagelkerke R^2^=0.22; Hosmer-Lemeshow p=0.38).

## DISCUSSION

This study demonstrates an elevated prevalence of NES among women with GDM (28.8%) compared with healthy pregnant controls (1.5%). NES clustered with poor sleep, severe fatigue, suboptimal lifestyle behaviors, lower physical activity, and reduced social support. To the best of our knowledge, this is among the first studies to concurrently characterize NES prevalence and its psychobehavioral correlates in a GDM cohort.

The 28.8% NES prevalence observed in our GDM cohort substantially exceeds the 2–6% commonly reported in general pregnant populations and approximates the high estimates documented among bariatric and severely obese cohorts^7^. Importantly, the increase in NES odds conferred by GDM persisted after adjustment for BMI, sleep, fatigue, and lifestyle factors, suggesting that GDM exerts an independent contribution beyond shared adiposity-related risk. This association may reflect circadian misalignment: altered glucose rhythms in GDM, combined with pregnancy-related shifts in melatonin secretion, cortisol patterning, and appetite-regulatory peptides, may amplify pre-existing vulnerabilities to disordered nocturnal eating^9,10^. The psychological burden of a GDM diagnosis—dietary restriction, glucose self-monitoring, and fetal-wellbeing anxiety—may further precipitate compensatory nocturnal intake^11^.

The magnitude of psychobehavioral impairment in NES-positive women is noteworthy. Effect sizes for fatigue, sleep quality, and lifestyle behaviors were large by conventional criteria^21^, indicating that NES rarely occurs in behavioral isolation. This pattern is consistent with the biobehavioral model proposed by Stunkard et al.^22^, in which NES represents a circadian-linked disruption of sleep–wake and feeding–fasting cycles with downstream consequences for energy regulation, mood, and adaptive health behavior. The universally poor sleep quality and the higher rate of severe fatigue in NES-positive women plausibly compromise GDM self-management, including dietary adherence and glucose monitoring^4^.

Higher HLBS and physical activity scores were independently protective against NES. These findings suggest that clinically meaningful behavioral improvements could translate into substantial reductions in NES risk. This observation is consistent with prior evidence that structured lifestyle interventions mitigate disordered eating and improve metabolic outcomes^23^, supporting the integration of multidomain behavioral assessment into routine antenatal GDM care.

The independent association between NES and NICU admission represents one of the most clinically relevant findings. This relationship remained significant after adjustment for maternal covariates, suggesting that NES contributes to neonatal morbidity through pathways not fully explained by conventional metabolic risk factors. Possible mechanisms include impaired maternal glycemic control related to nocturnal caloric intake and circadian feeding misalignment^9^, reduced treatment adherence potentially linked to fatigue and poor sleep^5^, and altered placental nutrient transfer from caloric redistribution to nighttime hours—a possibility indirectly supported by the somewhat lower 1-min Apgar scores. Notably, gestational age at birth did not differ significantly between groups, suggesting that the higher rate of NICU admissions may reflect maternal behavioral and metabolic factors affecting neonatal transitional physiology rather than prematurity alone.

### Strengths and limitations

Strengths include a relatively large sample with a defined control group, and comprehensive validated psychometric assessment. To the best of our knowledge, this is among the first studies to concurrently characterize NES prevalence, its psychobehavioral correlates, and its independent association with neonatal outcomes in a well-defined GDM cohort. Several limitations warrant acknowledgment. First, the cross-sectional design precludes causal inference, and reverse causation cannot be excluded. Second, NES was ascertained by self-report rather than structured clinical interview, although the NEQ has well-established psychometric properties^15^. Third, the single-center setting may limit generalizability. Finally, data regarding HbA1c and continuous glucose monitoring parameters, detailed macronutrient intake, depressive symptomatology, and obstetric complications were unavailable, which may have influenced findings.

## CONCLUSION

NES affected nearly one in three women with GDM compared with only 1.5% of healthy pregnant controls, and GDM was independently associated with an increased likelihood of NES. NES clustered with universally poor sleep, severe fatigue, suboptimal lifestyle behaviors, reduced physical activity, and lower social support, and independently predicted an increased risk of neonatal morbidity. Healthy lifestyle behaviors and physical activity emerged as modifiable protective factors. These findings suggest that routine NEQ-based screening deserves further prospective evaluation to determine its utility in identifying NES as a candidate target for behavioral interventions aimed at improving maternal and neonatal outcomes.

## Data Availability

The datasets generated and/or analyzed during the current study are available from the corresponding author upon reasonable request.
